# Organization of the *cpe* Locus in CPE-Positive *Clostridium perfringens* Type C and D Isolates

**DOI:** 10.1371/journal.pone.0010932

**Published:** 2010-06-03

**Authors:** Jihong Li, Kazuaki Miyamoto, Sameera Sayeed, Bruce A. McClane

**Affiliations:** 1 Department of Microbiology and Molecular Genetics, University of Pittsburgh School of Medicine, Pittsburgh, Pennsylvania, United States of America; 2 Department of Microbiology, Wakayama Medical University School of Medicine, Wakayama, Japan; University of California Merced, United States of America

## Abstract

*Clostridium perfringens* enterotoxin (encoded by the *cpe* gene) contributes to several important human, and possibly veterinary, enteric diseases. The current study investigated whether *cpe* locus organization in type C or D isolates resembles one of the three (one chromosomal and two plasmid-borne) *cpe* loci commonly found amongst type A isolates. Multiplex PCR assays capable of detecting sequences in those type A *cpe* loci failed to amplify products from *cpe-*positive type C and D isolates, indicating these isolates possess different *cpe* locus arrangements. Therefore, restriction fragments containing the *cpe* gene were cloned and sequenced from two type C isolates and one type D isolate. The obtained *cpe* locus sequences were then used to construct an overlapping PCR assay to assess *cpe* locus diversity amongst other *cpe*-positive type C and D isolates. All seven surveyed *cpe*-positive type C isolates had a plasmid-borne *cpe* locus partially resembling the *cpe* locus of type A isolates carrying a chromosomal *cpe* gene. In contrast, all eight type D isolates shared the same plasmid-borne *cpe* locus, which differed substantially from the *cpe* locus present in other *C. perfringens* by containing two copies of an ORF with 67% identity to a transposase gene (COG4644) found in Tn*1546*, but not previously associated with the *cpe* gene. These results identify greater diversity amongst *cpe* locus organization than previously appreciated, providing new insights into *cpe locus* evolution. Finally, evidence for *cpe* gene mobilization was found for both type C and D isolates, which could explain their *cpe* plasmid diversity.

## Introduction

*Clostridium perfringens* is an important pathogen of humans and domestic animals. The virulence of this organism is largely attributable to its producing at least 16 different potent toxins, although individual *C. perfringens* isolates never express this entire toxin arsenal [1], [2]. This characteristic is exploited by a commonly-used classification system assigning *C. perfringens* isolates to one of five types (A–E) based upon their production of four typing toxins. While all *C. perfringens* types make alpha toxin, type B isolates also express both epsilon toxin and beta toxin, type C isolates also produce beta toxin, type D isolates also make epsilon toxin and type E isolates also express iota toxin [1], [2].

Besides those typing toxins, *C. perfringens* isolates often produce one or more additional toxins. Notably, about 1–5% of all *C. perfringens* isolates produce a toxin named *C. perfringens* enterotoxin (CPE) [1], [3]. When expressed by type A isolates, CPE causes the gastrointestinal symptoms of the second most commonly-identified bacterial foodborne illness in the USA, ranking only behind *Salmonella* gastroenteritis [1], [2], [4]. About 75–80% of all type A food poisoning isolates carry their enterotoxin gene (*cpe*) on the chromosome [5], [6], [7], [8], [9], [10]. The chromosomal *cpe* locus present in most type A food poisoning isolates is highly conserved and includes an upstream IS*1469* sequence and flanking IS*1470* sequences [11], [12].

CPE-producing type A isolates also cause nonfoodborne human gastrointestinal (GI) diseases such as sporadic diarrhea or antibiotic associated diarrhea [13], [14]. Those type A nonfoodborne human GI disease isolates typically possess a plasmid-borne *cpe* gene [9], [15]. Two *cpe* plasmid families have been identified amongst most *cpe*-positive type A isolates [11], although rare type A soil isolates carry atypical *cpe* plasmids that have not yet been characterized [16]. The two major *cpe* plasmid families share a conserved region, corresponding to ∼50% of each plasmid [11], that includes a *tcp* locus closely resembling the *tcp* locus proven to mediate the conjugative transfer of *C. perfringens* tetracycline resistance plasmid pCW3 [17]. Carriage of this *tcp* locus likely explains the demonstrated conjugative transfer of the *cpe* plasmid from type A isolate F4969 [18].

The first of the two major *cpe* plasmid families of type A isolates, represented by the prototype plasmid pCPF5603, includes *cpe* plasmids that are typically ∼75 kb in size and also carry the *cpb2* gene encoding beta2 toxin [11], [13]. As discussed later, the *cpe* locus of these pCPF5603-like plasmids includes a *cpe* gene flanked by an upstream IS*1469* sequence and a downstream IS*1151* sequence [9], [11]. The second major *cpe* plasmid family, represented by the prototype *cpe* plasmid pCPF4969, includes *cpe* plasmids that are usually ∼70 kb in size and carry bacteriocin genes, but no *cpb2* gene [9], [11]. The *cpe* locus in the pCPF4969-like plasmids is flanked by an upstream IS*146*9 sequence and also contains, rather than the downsteam IS*1151* sequence found in the *cpe* locus of pCPF5603-like plasmids, a IS*1470-*like sequence downstream of the *cpe* gene [9], [11]. Some evidence suggests that the insertion sequences flanking the *cpe* gene of type A isolates may mobilize these toxin genes via formation of circular transposition intermediates [19].

Type E isolates typically carry plasmid-borne *cpe* sequences immediately downstream of their iota toxin genes [20], [21], but those *cpe* sequences are silent. This loss of CPE expression in type E isolates likely involves insertion of a mobile genetic element carrying the iota toxin genes near the *cpe* promoter, thereby blocking *cpe* transcription [20]. Flanking IS*1151*-like sequences present in the iota toxin locus may help to mobilize the iota toxin genes and, sometimes, the adjacent silent *cpe* sequences of type E isolates [21]. The iota toxin plasmids of type E isolates are often related to the major *cpe* plasmid families found in type A isolates, suggesting a common evolutionary origin [21]. However, the iota toxin plasmids are very large (>100 kb) due, in part, to their common carriage of lambda toxin genes and urease genes that are missing from *cpe* plasmids of type A isolates [20], [21].

In two recent surveys, ∼15% of 45 type C animal or human isolates and ∼25% of 39 type D animal disease isolates tested *cpe-*positive [22], [23]. Many of those isolates were shown to express CPE during sporulation [22], [23], which is consistent with suggestions that CPE may, at minimum, contribute to some cases of human enteritis necroticans caused by type C isolates [24]. However, the organization of the *cpe* locus in these type C and D isolates has not yet been studied. Therefore, the goal of the current study was to explore the relationship, if any, between the *cpe* locus of *cpe*-positive type A isolates vs. the *cpe* locus found in *cpe*-positive type C and D isolates.

## Materials and Methods

### Bacterial strains, media, and reagents

This study examined four *cpe*-positive type A isolates, seven *cpe*-positive type C isolates, eight *cpe*-positive type D isolates and two type E isolates carrying *cpe* sequences, as listed in Table 1. The toxin genotypes of these isolates had been determined previously using a toxin typing gene-specific multiplex PCR assay [22], [23]. Isolates were stored frozen in cooked-meat medium (Oxoid, Basingstock, England) or glycerol stocks. All isolates were grown overnight at 37°C in either FTG medium (fluid thioglycolate; Difco Laboratories, Michigan) or TGY medium (3% tryptic soy broth [Becton Dickinson and Company, Maryland], 2% glucose, 1% yeast extract [Difco], and 0.1% sodium thioglycolate [Sigma Chemical, Missouri]).

**Table 1:** Bacterial strains used in this study.

Strain	Type	Sources and date	*cpe* loci (plasmid size)	XbaI cut size
SM101	A	Food poisoning	C	5.1 kb
NCTC8239	A	Food poisoning	C	5.1 kb
F4969	A	GI disease	P (73 kb)	8.3 kb
F5603	A	GI disease	P (75 kb)	6.6 kb
CN5388	C	Human pigbel	P (90 kb)	6.5 kb
CN2076	C	Zeissler, UK, 1948	P (110 kb)	2.9 kb
CN2078	C	Zeissler, UK, 1948	P (75 kb)	2.9 kb
CN3758	C	Zeissler, UK, 1955	P (75 kb)	2.9 kb
CN3763	C	Zeissler, UK, 1955	P (110 kb)	2.9 kb
CN3753	C	Zeissler, UK, 1955	P (85 kb)	2.9 kb
CN3748	C	Zeissler, UK, 1955	P (75 kb)	2.9 kb
CN1183	D	Lamb, UK, 1942	P (75 kb)	5.0 kb
CN3842	D	Ewe, Spain, 1955	P (85 kb)	5.0 kb
CN4003	D	Lamb, unknown, 1955	P (110 kb)	5.0 kb
CN3948	D	Sheep, Teheran, 1956	P (110 kb)	5.0 kb
JGS1902	D	Sheep, enterotoxemia, USA, 1999	P (110 kb)	5.0 kb
JGS4138	D	Goat, sudden death, USA, 2002	P (110 kb)	5.0 kb
JGS4139	D	Goat, sudden death, USA, 2002	P (110 kb)	5.0 kb
JGS4152	D	Lamb, pulpy kidney, USA, 2002	P (110 kb)	5.0 kb
853	E	Calf with enteritis, North America	P (100 kb)	7.1 kb
NCIB10748	E	Calf with enteritis, North America	P (135 kb)	7.1 kb

### Pulsed-field gel electrophoresis (PFGE) and Southern blot analyses

Plugs of *C. perfringens* DNA were prepared as described previously [11], [21], [25]. Briefly, selected isolates (CN2078, CN5388, CN1183, CN4003, 853, NCIB107481, F5603 and F4969) were grown overnight in FTG broth at 37°C. A 0.1 ml aliquot of each FTG culture was then inoculated into separate 10 ml tubes of TGY broth and grown overnight at 37°C. The overnight TGY cultures were washed with TES buffer, pelleted, and resuspended in 200 µl of TE buffer. A 200 µl aliquot of 2% pulsed-field gel electrophoresis (PFGE)-certified agarose (Bio-Rad Laboratories, California) was then added to the resuspended cells, for a final agarose concentration of 1%.

These plugs were then electrophoresed in a CHEF-DR II PFGE system (Bio-Rad Laboratories) in 0.5× Tris-borate-EDTA buffer (Bio-Rad Laboratiories) at 14°C. The running parameters were: initial pulse, 1 sec; final pulse, 25 sec; voltage, 6 V/cm, 24 h. Mid-Range PFGE markers (New England Biolabs) were used as molecular size markers. After PFGE, the gel was stained with ethidium bromide, washed with distilled water, and photographed.

Digoxigenin (DIG)-labeled *cpe* probes were constructed, as described previously [11], [21], [25], with a PCR DIG probe synthesis kit (Roche, New Jersey) and internal *cpe* ORF primers. After hybridization of the *cpe* probe, performed as described previously [11], the pulsed-field gel Southern blots were developed using reagents from the DIG labeling and detection kit (Roche).

### Multiplex PCR genotyping analysis comparing *cpe* locus organization in *cpe*-positive type C or D isolates versus *cpe*-positive type A isolates

For these multiplex PCR reactions, template DNA was obtained, as described previously [26], from colony lysates of *cpe*-positive *C. perfringens* type A, C, and D isolates or from type E isolates carrying silent *cpe* sequences. Each PCR mixture contained 2 µl of template DNA, 10 µl of TAQ Complete 2× mix (New England Biolabs), and 1 µl of six multiple primers mix (final concentrations of 1 µM each for primers cpe4F, IS1470R1.3, IS1470-likeR1.6, and IS1151 and 0.2 µM each for primers 3F and 4R). The sequences of these primers have been reported previously [26]. Primers 3F and 4R amplify a product of ∼0.6 kb from internal *cpe* sequences; primers cpe4F and IS1470-likeR1.6 amplify a product of ∼1.6 kb from the *cpe* locus containing IS*1470-like* sequences, as found in pCPF4969-like plasmids of type A isolates; primers cpe4F and IS1151R0.8 amplify a product of ∼0.8 kb from the *cpe* locus containing IS*1151* sequences, as found in pCPF5603-like plasmids of type A isolates; and primers cpe4F and IS1470R1.3 amplify a product of ∼1.3 kb from the chromosomal *cpe* locus of type A isolates.

Each reaction mixture was subjected to the following PCR amplification conditions: cycle 1, 94°C for 2 min; cycles 2 through 40, 94°C for 30 sec, 61°C for 30 sec, and 68°C for 90 sec; with a final extension for 8 min at 68°C. An aliquot (20 µl) of each PCR sample was electrophoresed on a 1.5% agarose gel and then visualized by staining with ethidium bromide.

### Restriction fragment length polymorphism (RFLP) Southern blot analyses

Using the MasterPure gram-positive DNA purification kit (Epicentre, Wisconsin), *C. perfringens* DNA was isolated from *cpe*-positive type A strains F4969, F5603, SM101 and NCTC8239; *cpe*-positive type C strains CN3758, CN3753, and CN5388; *cpe-*positive type D strains CN1183, CN3842, CN4003, CN3948, JGS1902 and JGS4152; or silent *cp*e sequence-carrying type E strain NCIB10748. Each isolated DNA sample was then digested overnight with XbaI according to the manufacturer's (New England Biolabs) instructions. The digested DNA samples were electrophoresed on a conventional 1% agarose gel. The separated DNA digestion products were then transferred onto a nylon membrane (Roche) for hybridization with a *cpe* probe, as described above.

### Sequencing of the *cpe* ORF in representative type C and D strains

DNA was isolated from *cpe-*positive type C strains CN2078 and CN5388, or from *cpe*-positive type D strains JGS1902, JGS4139, CN1183, and CN4003, using the Master-Pure gram-positive DNA purification kit (Epicentre). PCR was then performed using Taq DNA polymerase from New England Biolabs and primers cpeF (5′-atgcttagtaacaatttaaatc-3′) and cpeR (5′-ttaaaatttttgaaataatattg -3′). The PCR reaction was performed in a Techne thermocycler (Techne, Germany) using the following conditions: 94°C for 2 min; 35 cycles of 94°C for 30 sec, 55°C for 40 sec, and 68°C for 1 min; with a single extension at 68°C for 5 min. The resultant 960 bp PCR products were then cloned into Topo® 2.1 vector (Invitrogen, California), and this insert was then sequenced at the University of Pittsburgh Core Sequencing facility. Results from these sequencing analyses are located in GenBank under accession numbers GQ225713, GQ225714, GQ225715, GQ225717, GQ225718, and GQ225719.

### Sequencing of the *cpe*-carrying XbaI fragments in type C and D isolates

DNA was isolated from *cpe*-positive type C strains CN2078 and CN5388, or from *cp*e-positive type D strain CN4003, as described above. A 2.5 µg aliquot of each isolated DNA sample was then digested overnight with XbaI according to the manufacturer's (New England Biolabs) instructions. The digested DNA samples were electrophoresed on a conventional 1% agarose gel. Bands were cut from that agarose gel based upon RFLP Southern blot results, gel purified, and cloned into the Topo® 2.1 vector (Invitrogen). The primers cpeF and cpeR were used to perform colony PCR to identify clones carrying *cpe* inserts. Plasmids were extracted from the PCR-positive colonies using the Qiagen plasmid preparation kit. Inserts present in the extracted plasmids were sequenced at the University of Pittsburgh core sequencing facility, using the primers listed in Table 2, 3 and 4.

**Table 2:** Primers sequence using in type C CN2078 *cpe* loci sequencing and overlap PCR.

Primers name	Sequence	Product size
dcmF	5′-gtaatccaggtagcagaaag-3′	642 bp (R1)
dcm2Rseq	5′-catttttatcttttctacgtgg-3′	
dcm2Fseq	5′-ccacgtagaaaagataaaaatgc-3′	996 bp (R2)
dcm3Rseq	5′-gtccgccagccgcatacttc-3′	
dcm3Fseq	5′-gaagtatgcggctggcggac-3′	674 bp (R3)
dcm4Rseq	5′-gttcaatttgatattgcaatttagaag-3′	
dcm4F	5′-cttctaaattgcaatatcaaattgaac-3′	1265 bp (R4)
dcmRseq	5′- tcacccaacaagtaactataatg-3′	
dcm5F	5′-tcattatagttacttgttgggtg-3′	1386 bp (R5)
cpeMF	5′-tccatcacctaaggactgttctaa-3′	
cpeMR	5′-ttagaacagtccttaggtgatgga-3′	1499 bp (R6)
p4111R	5′- cttaattgtaaaatgaaattgaac-3′	
p4112F	5′- aattctattaatgtaaaattctcc-3′	1040 bp (R7)
p5162R	5′- aacattttaataaacactcagttg-3′	
p5165F	5′- tctaaagattgtttagatagatg-3′	825 bp (R8)
p5990R	5′- tttcaaaatttttcaatagaattg-3′	

**Table 3:** Primers sequence using in type C CN5388 *cpe* loci sequencing and overlap PCR.

Primers name	Sequence	Product size
dcmF	5′-gtaatccaggtagcagaaag-3′	684 bp (R1)
61466	5′-ctacgtggaaatgttaaatctaagaac-3′	
61644R	5′-gttcttagatttaacatttccacgtag-3′	1052 bp (R2)
60619	5′-catactacctacgttgcatcttaagacgcttaaattag-3′	
60619R	5′-ctaatttaagcgtcttaagatgcaacgtaggtagtatg-3′	1024 bp (R3)
59620	5′-gagatatccgttaaacagatcaagttg-3′	
59620R	5′-caacttgatctgtttaacggatatctc-3′	1064 bp (R4)
cpeMF	5′-tccatcacctaaggactgttctaa-3′	
cpeMR	5′-ttagaacagtccttaggtgatgga-3′	1024 bp (R5)
5388over2	5′-gcctatattactaatgtacctag-3′	
5388seqF2	5′-ctaggtacattagtaatataggc-3′	1241 bp (R6)
5388overn3	5′-tttaatgcagctctgaatcatgg-3′	

**Table 4:** Primers sequence using in type D CN4003 *cpe* loci sequencing and overlap PCR.

Primers name	Seqence	Product size
1027F1	5′-ggatggctctataaatagacac-3′	615 bp (R1)
1027overR1	5′-tgtgctctagacatagcatcatc-3′	
1027upR1	5′-gttcttagatttaacatttccacgtag-3′	1015 bp (R2)
1027upNF3	5′-catactacctacgttgcatcttaagacgcttaaattag-3′	
1027overF2	5′-ctaatttaagcgtcttaagatgcaacgtaggtagtatg-3′	717 bp (R3)
1027upNF2	5′-gagatatccgttaaacagatcaagttg-3′	
1027overF3	5′-caacttgatctgtttaacggatatctc-3′	1343 bp (R4)
cpeMF	5′-tccatcacctaaggactgttctaa-3′	
cpeMR	5′-ttagaacagtccttaggtgatgga-3′	927 bp (R5)
1027overR2	5′-ctatcaataactttaactttttatac-3′	
1027seqR2	5′-gtataaaaagttaaagttattgatag -3′	692 bp (R6)
1027overR3	5′-gaacttgcaactttaaataattgc -3′	
1027seqR3	5′-tgcaattatttaaagttgcaagttc -3′	992 bp (R7)
1027overR4	5′-gccatttcctccccacttatc-3′	

### Sequencing of the *dcm* to *cpe* region in type C isolate CN2078

DNA was isolated from *cpe*-positive type C strain CN2078 as described above. PCR was then performed using the Long Range Taq DNA polymerase from New England Biolabs and primers dcmF and cpeseqMR (table 2). The PCR reaction was performed in a Techne thermocycler (Burkhardstdorf, Germany) and used the following conditions: 95°C for 2 min; 35 cycles of 95°C for 30 sec, 55°C for 40 sec, and 65°C for 5 min; with a single extension at 65°C for 10 min. The resultant 4 kb PCR product was cloned into the Topo® 2.1 vector. The plasmid insert was then sequenced at the University of Pittsburgh core sequencing facility using the primers listed in Table 2.

### Sequencing of the region upstream of the *cpe* gene in type D isolate CN4003

DNA was isolated from *cpe*-positive type D strain CN4003 as described above. A 2.5 µg aliquot of each isolated DNA sample was then digested overnight with EcoRI and KpnI, according to the manufacturer's (New England Biolabs) instructions. The digested DNA samples were electrophoresed on a conventional 1% agarose gel. The separated DNA digestion products were then transferred onto a nylon membrane (Roche) for hybridization with a *cpe* promoter probe, which was prepared using DIG labeled the PCR product of cpe-pro-F (5′-gcttaactattcttgatagttatct-3′) and cpe-pro-R (5′-gcattttcgaacaccattggattt-3′) as described above. Bands were cut from that agarose gel according to sizes determined by *cpe* Southern blot analyses (see Results), gel purified, and cloned into the Topo 2.1® vector (Invitrogen). The primers cpeupF and cpeupR were used to perform colony PCR to identify clones carrying a *cpe* promoter insert. Plasmids were extracted from the PCR-positive colonies using the Qiagen plasmid preparation kit. Inserts present in the extracted plasmids were sequenced at the University of Pittsburgh core sequencing facility using the primers listed in Table 4.

### Nucleotide sequence accession numbers for *cpe* locus sequences

Results from sequencing analyses of the *cpe* locus of type C isolates CN5388 and CN2078, or the type D CN4003 *cpe* locus sequence, are deposited in GenBank under accession numbers GQ225714, GQ225715 and GQ225713, respectively.

### Overlapping PCR analyses to evaluate *cpe* locus diversity amongst type C or D *cpe*-positive isolates

For these short-range PCRs, template DNA was obtained from *C. perfringens* colony lysates as described previously [11]. Each PCR mixture contained 2 µl of template DNA, 10 µl of TAQ Complete 2× mix (New England Biolabs), and 1 µl of each primer pair (1 µM final concentration). To compare the organization of the *cpe* locus present amongst different type C *cpe*-positive isolates, PCRs were performed that used overlapping primers for adjacent ORFs present in the *cpe* locus of either CN2078 (table 2) or CN5388 (table 3). These primers spanned from the *dcm* ORF in each *cpe* locus to the IS*1151-like* ORF downstream of the *cpe* ORF. For type D *cpe*-positive isolates, the overlapping PCRs were performed from the first transposase ORF upstream of the *cpe* gene to 2500 bp downstream of the *cpe* gene; primers are listed in Table 4. The design of these primers was based upon sequencing results obtained from the *cpe* locus of CN2078 (type C), CN5388 (type C) and CN4003 (type D), as determined above. The reaction mixtures, with a total volume of 20 µl, were placed in a thermocycler (Techne) and subjected to the following amplification conditions: one cycle of 95°C for 2 min; 35 cycles of 95°C for 30 sec, 55°C for 40 sec, and 68°C for 100 sec; and a single extension at 68°C for 10 min. PCR products were electrophoresed on a 1% agarose gel, which was then stained with ethidium bromide for product visualization.

### PCR identification of possible circular transposition intermediates carrying the *cpe* ORF

Each PCR mixture contained 5 µl of template DNA, which was a freshly prepared lysate from an overnight BHI agar culture of *cpe*-positive type C isolate CN2078, or *cpe*-positive type D isolate CN4003, 25 µl of TAQ complete 2× Master Mix (New England Biolabs), and 1 µl of each primer pair (1 µM final concentration). Primers used in these studies included dcmRseq, 1027upNF2 and cpeMR (table 2). PCR amplification were then performed in a Techne thermocycler using the following conditions: 95°C for 2 min; 35 cycles of 95°C for 30 sec, 54°C for 30 sec, and 68°C for 2 min; with a single extension of 68°C for 5 min. PCR products were separated on 1.5% agarose gels and visualized with ethidium bromide staining. PCR products were then excised from the gel using Quantum Prep freeze ‘N squeeze DNA gel extraction spin columns (Bio-Rad), cloned into pCR®2.1-TOPO vector, and sequenced at the University of Pittsburgh core sequencing facility.

## Results

### Pulsed-field gel Southern blot analyses of *cpe* location in type C isolates

**Figure 1:**
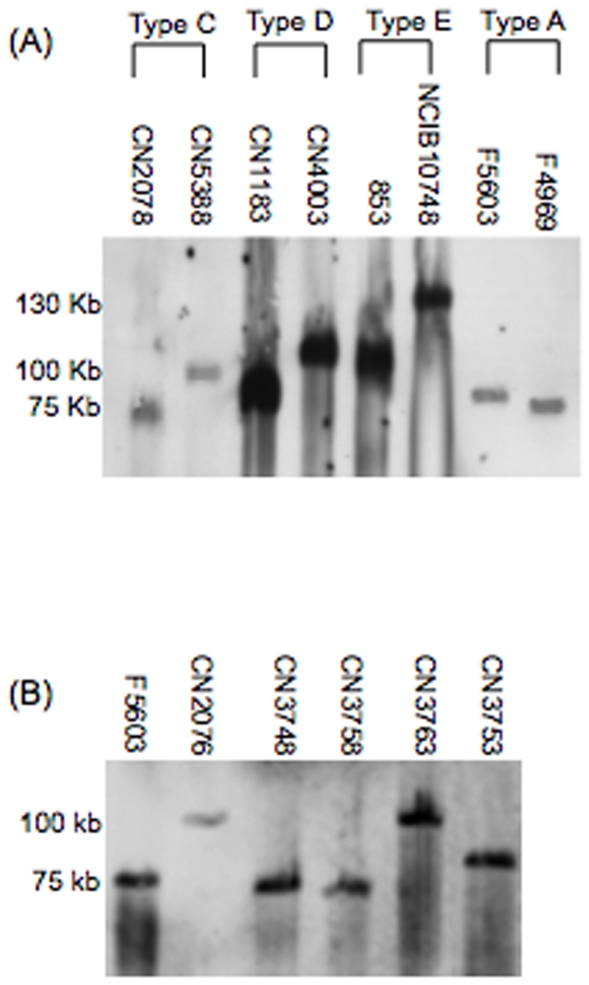
PFGE *cpe* Southern blot analyses of *cpe*-positive type A, C, D and E isolates. (A) DNA from type A, C, D or E isolates was subjected to PFGE prior to Southern blotting and hybridization with a DIG-labeled, *cpe*-specific probe. (B) DNA from type A (F5603) or type C (CN2076, CN3748, CN3758, CN3763 and CN3753) isolates was subjected to PFGE prior to Southern blotting and hybridization with a DIG-labeled, *cpe*-specific probe. The migration of molecular size markers is indicated on the left of the blot.

Using well-established conditions that allow plasmids (but not chromosomal DNA) to enter a pulsed-field gel and migrate according to their molecular sizes, previous studies [11], [21], [25], [27] had demonstrated that, i) some *cpe*-positive type A isolates carry a chromosomal *cpe* gene, while ii) other *cpe*-positive type A isolates and most, or all, *cpe*-positive type D isolates carry their *cpe* genes on large plasmids. Similarly, the silent *cpe* sequences of type E isolates are also carried by large plasmids [21]. However, inter- and intra- type differences have been observed in the size of plasmids carrying *cpe* genes or silent *cpe* sequences in type A, D and E isolates. Specifically, most *cpe* plasmids of type A isolates were found to be ∼70 kb or ∼75 kb in size [11], the *cpe* plasmids of type D isolates were shown to range in size from ∼75 kb to ∼110 kb [25], and the plasmids carrying silent *cpe* sequences in type E isolates were determined to vary in size from ∼100 kb to ∼135 kb [21]. A survey of type B isolates reported that these isolates rarely, if ever, are *cpe*-positive [28]. The current study first confirmed those previous reports of size differences in the plasmids carrying a *cpe* gene or silent *cpe* sequences amongst representative type A, D and E isolates (Fig. 1A, Table 1).

To our knowledge, the location (chromosomal vs. plasmid-borne) of the *cpe* gene in *cpe*-positive type C isolates has not yet been evaluated. Therefore, DNA from seven *cpe*-positive type C isolates was subjected to PFGE, followed by Southern blotting with a *cpe*-specific probe. As shown in Fig. 1A and 1B (and summarized in Table 1), this analysis localized the *cpe* gene of all surveyed type C isolates to plasmids, which ranged in size from 70–75 kb up to 110 kb. For comparison, Fig. 1A also shows type A isolates F5603 and F4969, which are known to carry *cpe* plasmids of 75 kb and 70 kb, respectively [11].

### Nucleotide sequencing of the *cpe* ORF in *cpe-*positive type C and D isolates

Having established that, as for *cpe*-positive type D isolates [25], the *cpe* gene is plasmid-borne in most, if not all, *cpe*-positive type C isolates, this study next investigated the here-to-fore unstudied *cpe* loci of *cpe*-positive type C and D isolates. This work initiated by sequencing the *cpe* ORF from two type C and four type D strains, which revealed that each of these isolates carries a *cpe* ORF nucleotide sequence that is identical to the highly conserved *cpe* ORF nucleotide sequence present amongst type A isolates [5], [29].

### Application of a multiplex PCR type A *cpe* locus subtyping assay to begin evaluating type C and D *cpe* locus organization

**Figure 2:**
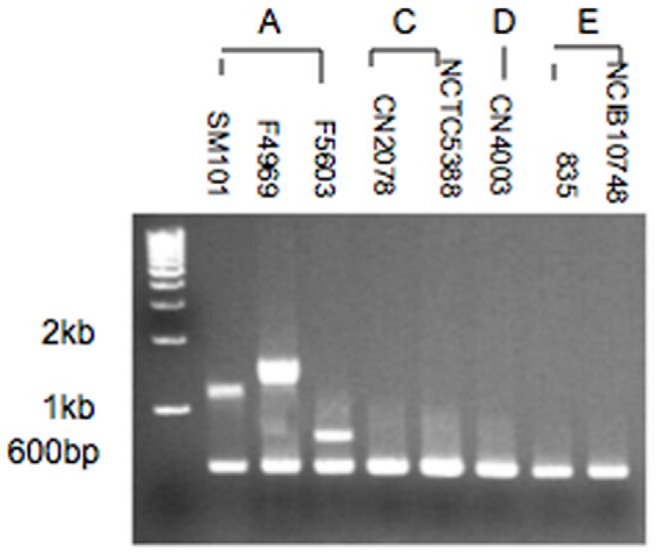
Analysis of *cpe* locus diversity in type C and D isolates using a multiplex PCR subtyping assay for *cpe* loci commonly found in type A isolates. Representative results obtained with this assay are shown for type A isolates known to carry a chromosomal *cpe* gene (SM101), a plasmid *cpe* gene with an associated IS*1470*-like sequence (F4969), or a plasmid *cpe* gene with an associated IS*1151* sequence (F5603). Also shown are representative results for this assay using culture lysates from *cpe*-positive type C isolates (CN2078, NCTC5388), *cpe*-positive type D isolates (CN4003) and type E isolates carrying silent *cpe* sequences (853 and NCIB10748). The migration of molecular size markers is indicated on the left of the blot.

This study next assessed whether the upstream and downstream sequences flanking the *cpe* gene in *cpe*-positive type C or D isolates resemble a characterized *cpe* locus found amongst *cpe*-positive type A isolates. This possibility was first evaluated using a previously described multiplex PCR assay [26] that is capable of distinguishing amongst the three characterized *cpe* loci commonly found in *cpe*-positive type A isolates (Fig. 2). As expected, this multiplex PCR assay correctly amplified an ∼0.6 kb internal *cpe* product using culture lysates of all three control type A *cpe* positive isolates. It also correctly amplified [26] an ∼0.8 kb product from culture lysates of type A isolate F5603, which carries an IS*1151* sequence downstream of its plasmid *cpe* gene; an ∼1.3 kb product from culture lysates of type A *cpe* positive isolate SM101, which carries a chromosomal *cpe* gene; and an ∼1.6 kb product from culture lysates of type A isolate F4969, which carries an IS*1470-like* sequence downstream of its plasmid *cpe* gene. Also consistent with previous studies [21], this multiplex PCR amplified the 0.6 kb internal *cpe* product, but no other products, from type E isolates carrying their plasmid-borne silent *cpe* sequences in a locus organized differently from those found in *cpe*-positive type A isolates.

Having confirmed the reliability of this multiplex PCR assay for differentiating amongst the three common *cpe* loci found amongst type A isolates, the assay was then applied to seven *cpe*-positive type C and eight *cpe*-positive D isolates. These analyses amplified the ∼0.6 kb internal *cpe* product from all surveyed isolates (Fig. 2 and data not shown), further confirming that these type C and D isolates are each *cpe*-positive. However, no other products were amplified from lysates of any surveyed *cpe*-positive type C or D isolates, suggesting that their *cpe* loci are not organized similarly as the type A chromosomal *cpe* locus, the pCPF4969-like *cpe* locus or the pCPF5603-like *cpe* locus.

### RFLP analyses of *cpe* locus heterogeneity amongst *cpe*-positive type C and D isolates

Fig. 2 results were consistent with the existence of organizational differences between the *cpe* loci found in type A isolates vs. the *cpe* loci found in the surveyed type C or D isolates. This suggestion was then further explored by RFLP analyses.

As reported previously [5], [10], [15], [30], the *cpe* gene localized (Fig. 2) to an ∼5.7 kb XbaI fragment in type A isolates, such as NCTC8239 and SM101, known to carry a chromosomal *cpe* gene. As also reported [10], [15], [30], the *cpe* gene was detected on larger XbaI fragments in type A isolates known to carry a plasmid-borne *cpe* gene, i.e., in type A isolates F5603 and F4969 the *cpe* gene localized to ∼6.6 kb or ∼8.3 kb XbaI fragments, respectively. Also consistent with previous sequencing and PCR analyses [21], these analyses showed that type E isolate NCIB10748 carries its silent *cpe* sequences on a 7.1 kb XbaI fragment (Table 1).

**Figure 3:**
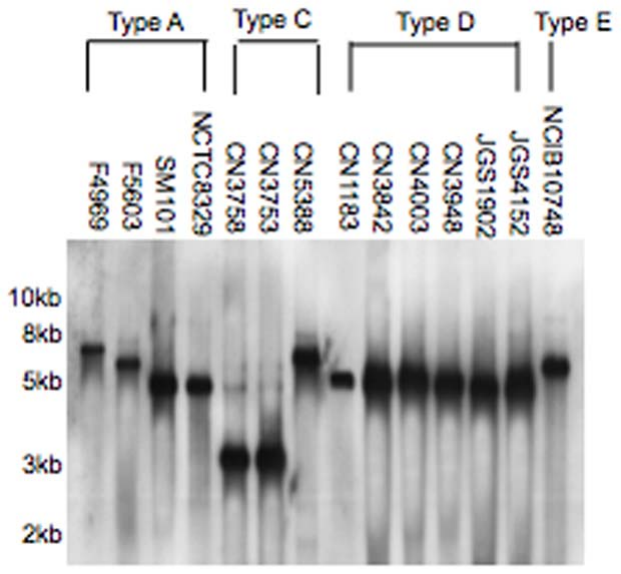
RFLP analyses of *cpe*-positive type A, C, and D isolates and type E isolates carrying silent *cpe* sequences. DNA from each isolate was digested with XbaI prior to conventional agarose gel electrophoresis and Southern blot hybridization with a *cpe*-specific probe. The migration of molecular weight markers is shown on the left of the blot.

When eight *cpe*-positive type D isolates were similarly surveyed by RFLP, no size diversity was noted amongst their *cpe*-carrying XbaI fragments, i.e., all of these isolates were found to carry their *cpe* gene on an ∼5 kb XbaI fragment. In contrast. the surveyed *cpe*-positive type C isolates showed limited heterogeneity in the size of their *cpe*-carrying XbaI fragments. Specifically, CN5388 carried *cpe* on an ∼6.5 kb XbaI fragment, while the other surveyed type C isolates all carried their *cpe* gene on an ∼3 kb XbaI fragment (Table 1 and Fig. 3).

### Sequencing of *cpe* loci in type C isolates

In combination, the Fig. 2 and 3 results suggested that the *cpe* locus is often organized differently between *cpe*-positive type C isolates versus *cpe*-positive type A or D strains or even amongst *cpe*-positive type C strains. Therefore, the ∼3 kb *cpe*-carrying CN2078 XbaI fragment and ∼6.5 kb *cpe*-carrying CN5388 XbaI fragment were sequenced. Because the short, ∼3 kb CN2078 XbaI fragment did not include *dcm*, which is usually located near the *cpe* gene in type A isolates [11], [13], a long range PCR reaction was performed to attempt linking of *dcm* to *cpe* using CN2078 strain DNA. A product of ∼4 kb was successfully obtained from this PCR and then sequenced.

**Figure 4:**
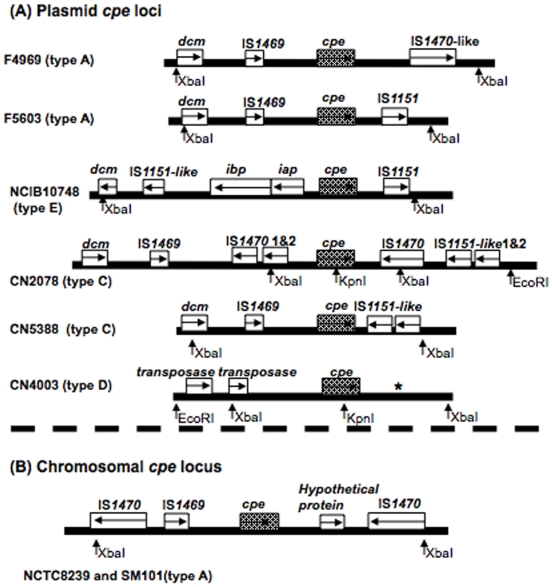
Organization of *cpe* loci in type A, C, D and E. A) Organization of plasmid *cpe* loci. B) Organization of the type A chromosome *cpe* locus. Each box represents an ORF. * indicates a region with sequence similarity to sequences present downstream of *cpe* in F4969, except for the absence of an IS*1470-like* gene. Sequences of *cpe* loci in F4969, F5603, NCIB10748, NCTC8239 and SM101 have been reported previously [11], [21], [38]. Sequences of CN2078, CN5388 and CN4003 are based upon results of this study. The arrows show predicted enzyme (EcoRI, XbaI and KpnI) cleavage sites used in this study.

As shown in Fig. 4, these sequencing analyses revealed that the CN2078 *cpe* locus bears some resemblance to the type A chromosomal *cpe* locus, i.e., the CN2078 *cpe* locus contains an IS*1469* and two IS*1470* sequences and it also has a *cpe* ORF situated between two IS*1470* genes. However, two differences were identified between the chromosomal *cpe* locus of type A isolate SM101 and the plasmid-borne *cpe* locus of type C isolate CN2078; i) the IS*1469* sequence present in the CN2078 *cpe* locus is situated differently with respect to the IS*1470* sequence present upstream of *cpe* and ii) the IS*1151*-like sequence located downstream of *cpe* in CN2078 is absent from the type A chromosomal *cpe* locus. Sequencing results for the ∼7 kb CN5388 XbaI fragment showed that this unusual (by RFLP analysis) type C *cpe* locus is missing the two copies of IS*1470* that are present in the *cpe* locus of CN2078.

### Overlapping PCR analyses to evaluate *cpe* locus diversity amongst type C *cpe*-positive isolates

**Figure 5:**
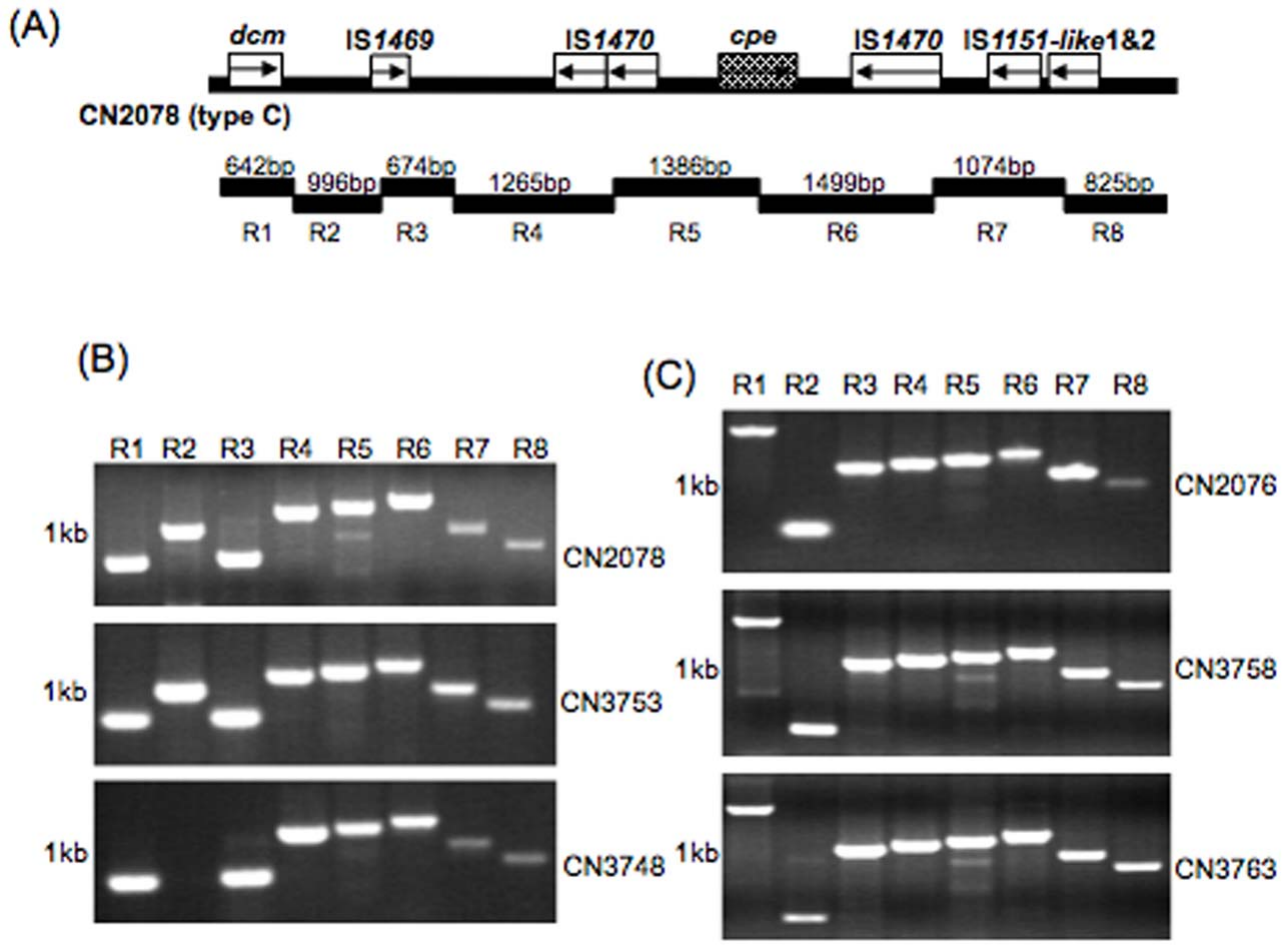
Overlapping PCR assay analysis of *cpe* locus diversity amongst type C isolates. An overlapping PCR assay specific for amplification of the type C isolate CN2078 *cpe* locus region (R1 to R8) was performed using the primer battery shown in Table 2. (A) Map depicting the relationship between CN2078 *cpe* locus ORFs and reactions comprising this overlapping PCR battery. (B) PCR products produced by these reactions using DNA from type C isolates: CN2078, CN3753 and CN3748. (C) PCR products produced by these reactions using DNA from type C isolates: CN2076, CN3758 and CN3763. Numbers at left of each gel indicate migration of size markers in kb.

Based upon the sequence obtained for the type C CN2078 *cpe* locus, an overlapping PCR assay (8 reactions) was developed to evaluate the presence of this *cpe* locus in other *cpe-*positive type C isolates. This assay was then applied to assess *cpe* loci diversity in six type C *cpe* positive isolates that, like CN2078, carry their *cpe* gene on an ∼2.9 kb XbaI fragment (Fig. 3 and Table 1). In this experiment, DNA from all six surveyed type C isolates supported full or partial amplification of the expected PCR products. In particular, DNA from type C isolates CN3753 and CN3748 gave exactly the same amplification pattern as was obtained using CN2078 DNA (Fig. 5). DNA from the other three isolates showed some amplification pattern differences for sequences upstream of the *cpe* gene, but supported conserved amplification of products corresponding to sequences immediately adjacent to, or downstream of, the *cpe* gene.

**Figure 6:**
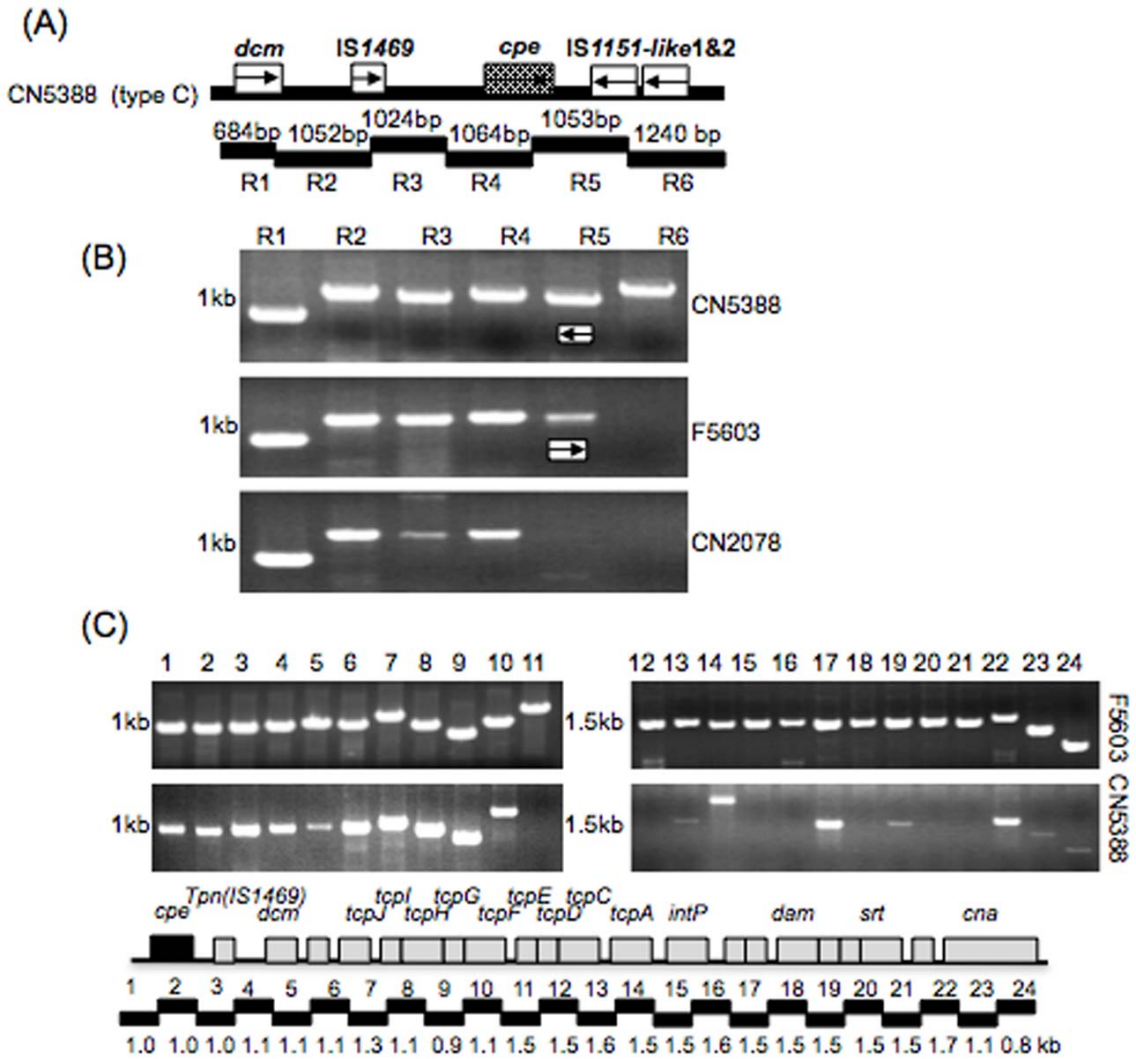
Overlapping PCR comparison of type C isolate CN5388 versus type A isolate F5603. (A) Map depicting the relationship between ORFs and reactions in the *cpe* locus overlapping PCR battery (reactions R1 to R6) was performed using the primer battery show in Table 3. (B) Products of these reactions amplified by PCR using DNA from type C isolate CN5388 and CN2078 or type A isolates F5603. Arrows indicate that IS*1151* sequences are oppositely oriented in CN5388 vs. F5603. (C) Products obtained when DNA from CN5388 or F5603 were subjected to a previously described [11] overlapping PCR assay specific for the conserved region of type A *cpe* plasmids pCPF5603 and pCPF4969. Numbers at left of each gel indicate migration of size markers in kb.

Sequencing had shown that CN5388 possesses a very different *cpe* locus from that found in the other surveyed type C isolates (Fig. 4) and also indicated that the CN5388 *cpe* locus sequence shares some resemblance to the plasmid borne *cpe* locus of pCPF5603 carried by type A isolate F5603 (Fig. 4). This finding was consistent with results obtained using an overlapping PCR assay based upon the CN5388 *cpe* locus sequence (Fig. 6A and B). Therefore, given their *cpe* locus similarity, it was possible that the CN5388 *cpe* locus might be present on a similar plasmid as pCPF5603. However, an overlapping PCR assay for the conserved region of pCPF5603 (and pCPF4969) amplified only the *tra* region from CN5388 DNA (Fig. 6C and data not shown).

### Sequencing of the *cpe* locus in type D isolate CN4003

Results from the Fig. 3 RFLP analyses demonstrated that all of the surveyed type D isolates possess a *cpe*-carrying XbaI fragment of the same ∼5 kb size. Therefore, the ∼5 kb XbaI fragment carrying the *cpe* gene of type D isolate CN4003 was cloned into the pPCR2.1®-TOPO vector and sequenced. Efforts to PCR link the *dcm* gene to *cpe* in type D isolates were unsuccessful (data not shown). Consequently, additional upstream sequence in the type D *cpe* locus was obtained by cloning and sequencing an ∼3 kb EcoR1/KpnI fragment containing sequences upstream of the XbaI site in the *cpe* locus of CN4003.

Together, these sequencing analyses revealed that CN4003 possesses a novel *cpe* locus organization different from that found in any other characterized *cpe*-positive *C. perfringens* (Fig. 4). Specifically, CN4003 was found to possess, upstream of its *cpe* gene, two copies of an ORF with 67% identity to a transposase gene (COG4644) found in Tn*1546*, but not previously associated with the *cpe* gene. This CN4003 *cpe* locus also has sequences found downsteam of the *cpe* gene in type A isolate F4969, except for the absence of an IS*1470-like* insertion sequence (Fig. 4).

### Overlapping PCR analyses to evaluate *cpe* locus diversity amongst type D *cpe*-positive isolates

**Figure 7:**
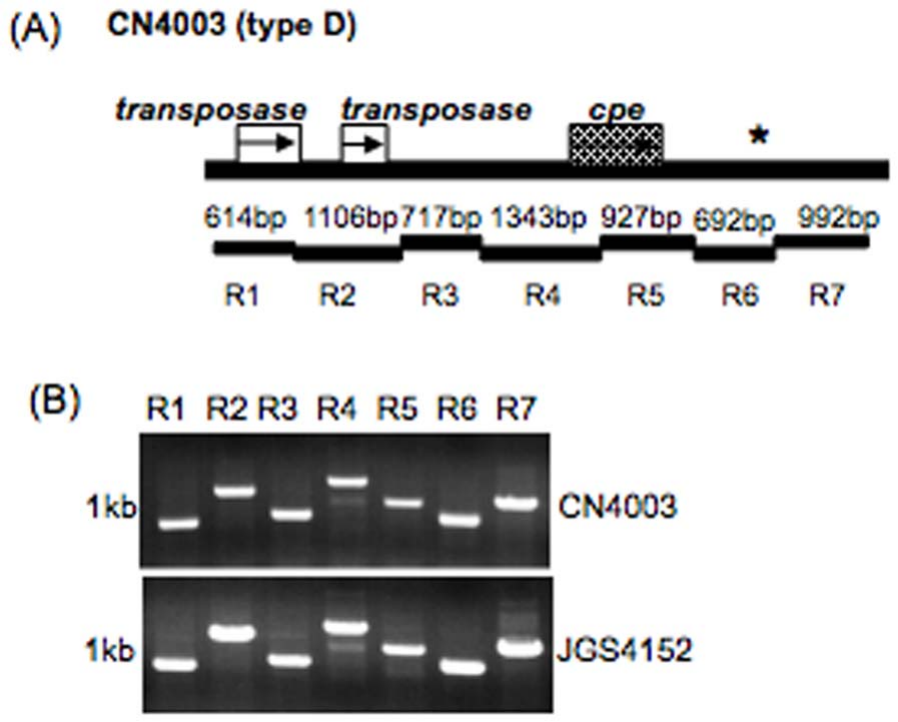
Overlapping PCR assay analysis of *cpe* locus diversity amongst type D isolates. An overlapping PCR assay specific for amplification of the type D isolate CN4003 *cpe* locus region (R1 to R7) was performed using the primer battery shown in Table 4. (A) Map depicting the relationship between ORFs and each reaction in this overlapping PCR battery. * indicates a region with sequence similarity to sequences downstream of *cpe* in F4969, except for the absence of IS*1470-like* gene. (B) Products of these reactions using DNA from two representative type D isolates: CN4003 and JGS1902. Numbers at left of each gel indicate migration of size markers in kb.

Based upon the sequence obtained for the type D CN4003 *cpe* locus, an overlapping PCR assay (7 reactions) was developed to specifically evaluate the presence of this *cpe* locus in other type D isolates. When this assay was applied to assess the diversity of *cpe* loci in seven other type D *cpe* positive isolates, the amplification pattern obtained was identical for each isolate (Fig. 7 and data not shown). This result strongly suggested that many, if not all, type D *cpe*-positive isolates share a very similar *cpe* locus, consistent with the Fig. 3 RFLP results.

### PCR identification of possible circular transposition intermediates carrying the *cpe* ORF

**Figure 8:**
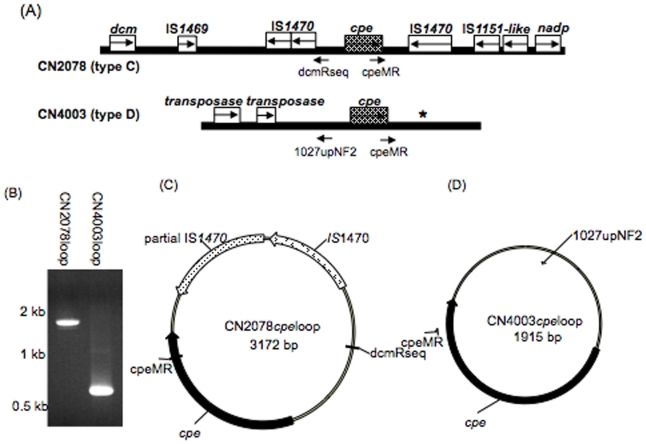
Detection of potential circular transposition intermediates carrying the *cpe* gene in type C and D isolates. (A) Diagram of the *cpe* locus in type C isolate CN2078 and type D isolate CN4003. (B) PCR amplification of *cpe-*containing circular intermediates using the primers dcmRseq and cpeMR with CN2078 DNA or primers 1027upNF2 and cpeMR with CN4003 DNA. (C) Diagram derived from sequencing the CN2078 loop product of panel B that was amplified using primers dcmRseq and cpeMR. Black regions of the circle correspond to the amplified product. (D) Diagram derived from sequencing the product from CN4003 loop product of pane B that was amplified using primers 1027upNF2 and cpeMR. Black regions of the circle correspond to the amplified product.

The results presented above indicated that the *cpe* gene present in many, if not all, type C and D isolates is closely associated with several different insertion sequences, including (for type D isolates) some not previously associated with the *cpe* gene. Since IS elements in type A isolates can apparently mediate excision and formation of possible *cpe*-containing circular transposition intermediates that might facilitate *cpe* gene mobilization [19], primers in opposite orientations were used in PCR reactions to evaluate whether similar *cpe*-containing circular intermediates might also form in *cpe*-positive type C and D isolates. Primers dcmRseq and cpemR consistently amplified a strong 1.7 kb PCR product from *cpe*-positive type C isolate CN2078. When this PCR product was sequenced, it corresponded to sequences containing *cpe*, one intact IS*1470* insertion sequence and one partial IS*1470* insertion sequence (Fig. 8). Similarly, PCR primers from all three surveyed type D *cpe* positive isolates amplified a strong 0.6 kb PCR product. Sequencing showed that this PCR product contains a partial *cpe* ORF and some sequence upstream of *cpe* but no insertion sequence (Fig. 8).

## Discussion

Except for the *cpb2* ORF encoding beta2 toxin [13], [31], the ORF sequences of most *C. perfringens* toxin genes are usually highly conserved from isolate-to-isolate. For example, only limited sequence diversity has been observed for the *cpa* (*plc*) ORF encoding alpha toxin, the *cpb* ORF encoding beta toxin, the ORFs of the *iap/ibp* genes encoding iota toxin, and the *etx* ORF encoding epsilon toxin [21], [22], [23], [32], [33]. Similarly, previous studies [5] had revealed that the *cpe* ORF sequence amongst surveyed type A isolates is invariant, regardless of whether this toxin gene is chromosomal or plasmid-borne. The current study now extends that earlier finding by showing that the *cpe* sequence is identical amongst type A, C and D isolates. This exceptional conservation of the *cpe* ORF sequence is particularly remarkable given the considerable diversity between sequences flanking the *cpe* gene in many type A, C and D isolates, as discussed below. Collectively, these observations might suggest that CPE protein functionality is intolerant of most mutations, causing selective pressure to maintain an invariant *cpe* ORF sequence amongst CPE-producing type A, C and D isolates. The single known exception to this pattern of invariant *cpe* sequences occurs with type E isolates, where a genetic element carrying the iota toxin gene has apparently inserted near the *cpe* promoter, silencing the *cpe* gene. Upon this silencing, a number of missense, nonsense and frame-shift mutations accumulated in the silent *cpe* ORF of type E isolates. Since the same mutations are present in the *cpe* sequences of most or all type E isolates [20], [21], this *cpe* silencing is thought to have occurred relatively recently [20]. One possibility is that acquisition of iota toxin genes may have compensated for the loss of a functional *cpe* gene by providing type E isolates a selective advantage in a new pathogenic niche, particularly since *cpe* expression occurs only during sporulation while iota toxin is produced by vegetative cells.

Previous studies have localized the *cpe* gene near a *dcm* gene on both the pCPF4969-like and pCPF5603-like plasmids of type A isolates, [9], [30]. The current study now demonstrates that a *dcm* gene is also proximal to the plasmid-borne *cpe* gene in many, if not all, type C isolates. One previously proposed [9] explanation for this strong association between *dcm* and *cpe* is that the *dcm* region of plasmids represent a hot-spot for insertion of certain mobile genetic elements, including some carrying a *cpe* gene. Consistent with this hypothesis, the *cpe* gene has now been localized near *dcm* in those *cpe* loci where the *cpe* gene is flanked by various combinations of IS*1469*, IS*1470*, IS*1470*-like, IS*1151* or IS*1151*-like sequences [9]. The possibility that the *dcm* region of *C. perfringens* plasmids represents a hot spot for insertion of mobile genetic elements consisting of certain IS elements and adjacent toxin genes receives further support from the established proximity of *dcm* to, i) plasmid-borne IS*1151*-*iota* toxin gene sequences in type E isolates and ii) plasmid-borne IS*1151*-*etx* sequences in type B and D isolates [21], [25], [27].

However, the current study may have also identified the first exception to the general association between *dcm*, insertion sequences, and plasmid-borne *C. perfringens* toxin genes. Specifically, attempts to PCR-link *dcm* and *cpe* proved unsuccessful in the surveyed *cpe*-positive type D isolates. If future studies confirm that *dcm* and *cpe* are not proximal in type D isolates, this could be explainable by our observation that the *cpe* gene of type D isolates is flanked by unique transposase sequences not previously associated with *C. perfringens* toxin genes. These transposase sequences share 67% identity to the transposase (COG4644) of Tn*1546*, which is a Tn*3*-related transposon commonly distributed amongst plasmids found in Gram-positive bacteria, including several *Bacillus* spp, *Staphylococcus aureus* and *Enterococcus faecium* [34]. Conceivably, these unique transposase sequences flanking the *cpe* gene in many, if not all, type D isolates may mobilize *cpe* and prefer integrating into other plasmid sequences rather than integrating near the *dcm* gene.

Experimental support for possible IS-mediated mobilization of adjacent toxin genes in *C. perfringens* has largely been provided by studies demonstrating that primers in opposite orientations support PCR amplification of toxin gene-containing circular DNAs, which may represent transposition intermediates [19], [21], [25], [28]. Prior to the current study, possible circular transposition intermediates had been detected that carry the *cpe* genes of type A isolates, the iota toxin genes of type E isolates, the *cpb-tpeL* genes of type B isolates and the *etx* genes of type D isolates [19], [21], [28], [35]. Results presented in the current study support the possibility that the *cpe* genes of many type C and D isolates, although often present in differently organized loci from those found in type A isolates, can also be mobilized by adjacent sequences to form possible circular transposition intermediates.

This putative mobilization of toxin genes by adjacent IS sequences may help to explain why the same *C. perfringens* toxin gene can be found on different plasmid backbones. For example, the *etx* gene is almost always localized on a ∼65 kb plasmid in type B isolates, yet only a minority of type D isolates carry that ∼65 kb *etx* plasmid [25]. Instead, type D isolates carry a diverse range of *etx* plasmids, some also carrying the *cpe* gene [25]. Since potential circular transposition intermediates carrying either the *cpe* or *etx* genes have now been identified (this study, [25]), it is possible that the toxin plasmid diversity of type D isolates reflects this mobility of toxin gene-carrying mobile genetic elements.

The major finding of the current study is the provision of new insights into the diversity of *cpe* loci found amongst *C. perfringens* isolates. All surveyed *cpe-*positive type D isolates were shown to carry the same plasmid-borne *cpe* locus. This conclusion holds for type D isolates previously shown [25] to carry *cpe* and *etx* on the same plasmid, as well as type D isolates that carry those two toxin genes on distinct plasmids. These observations could indicate that a similar mobile genetic element has mobilized this conserved *cpe* locus from a progenitor *cpe*-carrying plasmid present in a type D isolate onto other plasmids present in that same isolate or, after conjugative transfer, in other type D isolates.

With respect to type C isolates, the current study suggests that many of these isolates also share a relatively conserved *cpe* locus, although the *cpe* locus of CN5388 is more divergent since it lacks the IS*147*0 sequences that flank the *cpe* gene in the other surveyed type C isolates. The type C *cpe* locus variants identified in this study generally resemble the *cpe* loci found in type A isolates by sharing many of the same IS elements, although in different arrangements [11]. This may suggest a common evolutionary origin for the *cpe* loci of many type A and C isolates that is distinct from the *cpe* locus found in many type D isolates. Of particular note is the extensive similarity between the type A chromosomal *cpe* locus and the common plasmid-borne *cpe* locus present in CN2078 and most of the other surveyed type C isolates. One possible explanation for this similarity is that the chromosomal *cpe* locus of a type A isolate may have excised as a mobile genetic element and, after some recombination, integrated into a conjugative plasmid, which then transferred to a type C isolate. Alternatively, IS elements may have mobilized the plasmid-borne type C *cpe* locus so it could then integrate onto the *C. perfringens* chromosome, followed later by loss of the *cpb* plasmid to convert the isolate back to a type A isolate. If this second possibility is true, this chromosomal integration of a *cpe*-carrying mobile genetic element must have occurred rarely since most, if not all, chromosomal *cpe* type A isolates appear to be related, as assessed by MLST analyses [36], [37].

A final interesting observation from the current study is that the single variant *cpe* locus observed amongst the surveyed type C isolates involved an isolate causing human pigbel (enteritis necroticans). Although such clinical isolates are difficult to obtain, it would be interesting to evaluate whether other *cpe-*positive, type C pigbel isolates also carry this same variant *cpe* locus, possibly suggesting virulence significance or a common evolutionary origin. Additional clarification of these and other issues about *cpe* locus diversity and evolution are the subject of additional studies ongoing in our laboratory.
